# Scanning iron response regulator binding sites using Dap-seq in the *Brucella* genome

**DOI:** 10.1371/journal.pntd.0011481

**Published:** 2023-07-17

**Authors:** Huan Zhang, Tianhao Sun, Xudong Cao, Yifan Wang, Zhongchen Ma, Yueli Wang, Ningning Yang, Mingguo Xu, Xiaoyu Deng, Honghuan Li, Benben Wang, Jihai Yi, Zhen Wang, Qian Zhang, Chuangfu Chen

**Affiliations:** 1 School of Animal Science and Technology, Shihezi University, Shihezi City, Xinjiang, China; 2 Collaborative Innovation Center for Prevention and Control of High Incidence Zoonotic Infectious Diseases in Western China, Shihezi, Xinjiang, China; 3 School of Medicine, HeXi University, Zhangye City, Gansu, China; 4 State key Laboratory of Agricultural Microbiology/College of Veterinary Medicine Huazhong Agricultural University 1 Wuhan, China; 5 School of Life Science, Shihezi University, Shihezi City, Xinjiang, China; 6 State Key Laboratory for Sheep Genetic Improvement and Healthy Production, Xinjiang Academy of Agriculture and Reclamation Science,Shihezi, Xinjiang, China; Universidad de Costa Rica, COSTA RICA

## Abstract

Iron is an essential element required for all organisms. Iron response regulator (Irr) is a crucial transcriptional regulator and can affect the growth and iron uptake of *Brucella*. The growth rate of *Brucella melitensis* M5-90 *irr* mutant was significantly lower than that of *B*. *melitensis* M5-90 under normal or iron-sufficient conditions, however, the growth rate of the *B*. *melitensis* M5-90 *irr* mutant was significantly higher than that of *B*. *melitensis* M5-90 under iron-limited conditions. In addition, *irr* mutation significantly reduced iron uptake under iron-limited conditions. Previous studies suggested that the Irr protein has multiple target genes in the *Brucella* genome that are involved in iron metabolism. Therefore, in the present study, a Dap-seq approach was used to investigate the other iron metabolism genes that are also regulated by the Irr protein in *Brucella*. A total of seven genes were identified as target genes for Irr in this study and the expression levels of these seven genes was identified using qRT-PCR. The electrophoretic mobility shift assay confirmed that six out of the seven genes, namely *rirA* (BME_RS13665), *membrane protein* (BME_RS01725), *hypothetical protein* (BME_RS09560), *ftrA* (BME_RS14525), *cation-transporting P-type ATPase (zntA)* (BME_RS10660), and *2Fe-2S binding protein* (BME_RS13655), interact with the Irr protein. Furthermore, the iron utilization and growth assay experiments confirmed that *rirA* was involve in iron metabolism and growth of *Brucella*. In summary, our results identified six genes regulated by the Irr protein that may participate in iron metabolism, and the *rirA* was identified as a regulon of Irr and it also plays a role in iron metabolism of *Brucella*. Collectively, these results provide valuable insights for the exploration of *Brucella* iron metabolism.

## Author summary

Iron response regulator (Irr) plays a key role in iron metabolism of *Brucella*. In this study, the *Brucella melitensis* M5-90 *irr* mutant appeared significant different of growth and iron utilization rate compared with that of the *Brucella melitensis* M5-90 strain. However, the specific binding sites of Irr in *Brucella* are not fully understood. Here, we used Dap-seq to identify the potential target genes of Irr in the genome of *Brucella melitensis* M5-90. A total of seven genes were identified as target genes for Irr and the expression of levels of these seven genes were identified using qRT-PCR. In addition, Six out of seven genes namely *rirA*, *membrane protein*, *hypothetical protein*, *ftrA*, *zntA and 2Fe-2S binding protein* interact with the Irr protein. Furthermore, the mutation of *rirA* markedly affected the iron utilization and growth rate of *Brucella*. Overall, these results provide valuable insights for the exploration of *Brucella* iron metabolism.

## Introduction

*Brucella* is a Gram-negative, facultative intracellular pathogen that causes brucellosis, a worldwide zoonotic disease. Annually, more than 500,000 cases of human brucellosis occur globally [1]. This disease is characterized by undulant fever and chronic debilitating symptoms, such as endocarditis, spondylitis, arthritis, and meningitis; in livestock, it causes abortion and infertility [2–4]. Several studies have shown that *Brucella melitensis* is the predominant pathogen responsible for human or animal brucellosis in many provinces in China, including Xinjiang, Inner Mongolia, and Shanxi [4–12]. However, the pathogenic mechanisms of *Brucella* are not well understood. During the invasion of the host tissue, the organism invades and multiplies within professional and nonprofessional phagocytes and eventually establishes persistent infection in the host [13]. Metal metabolism plays a role in the expression of virulence genes required by *Brucella* to replicate or regulate the immune response in a host [14–19].

Iron is an indispensable micronutrient required by nearly all organisms as it is involved in many cellular processes, including energy metabolism, electron transport, and nucleotide biogenesis [20–24]. Although iron is available in the environment, it is scarce inside host cells to prevent oxidative damage to itself or replication of pathogens, and the change in environmental iron concentration is also an important signal for the induction of expression of virulence genes [22]. Hence, many pathogens have evolved acquisition mechanisms to obtain iron in the iron-restricted environment of mammalian cells. For example, bacteria obtain iron through the assimilation of heme or from iron-binding proteins by producing siderophores (a small molecule with an extremely high affinity for iron) when the intracellular iron concentration drops below a threshold [25]. Currently, two *Brucella* siderophores (2,3-dihydroxybenzoic acid (2,3-DHBA) and brucebactin) have been identified [26,27].

Iron response regulator (Irr) is a member of Fur family of transcriptional regulators that was first identified as a regulator of heme biosynthesis in *Bradyrhizobium japonicum* [28,29]. Previous studies have revealed that *B*. *abortus* Irr is also involved in heme biosynthesis and siderophore production under conditions of iron limitation. In addition, the ability of *B*. *abortus* to secrete brucebactin was significantly reduced when *irr* was knocked out [30,31]. A study has shown that *bhuA* is a crucial heme transporter, the *B*. *abortus bhuA* mutant cannot utilize heme as an iron resource *in vitro*, and Irr can regulate the expression of *bhuA* by directly binding to the promoter region of *bhuA* [32]. In addition, *Brucella* heme utilization oxygenase Q (*bhuQ*) was identified as a heme oxygenase in *B*. *abortus* 2308; it is cotranscribed with the iron-responsive regulator *rirA*, and both genes are regulated by Irr [33]. In addition to Irr, rhizobial iron regulator A (RirA) was first identified as an iron-responsive regulator in *Rhizobium leguminosarum* [34,35]. A previous study reported that RirA protein is active under iron-sufficient conditions, repressing iron uptake and the expression of *rirA* was regulated by Irr in *Agrobacterium tumefaciens* [36]. It can be inferred from these results that numerous genes associated with iron metabolism may exist in the *Brucella* genome and they might also be regulated by Irr. Therefore, the aim of this study is to screen the other iron metabolism genes regulated by Irr in *Brucella* and to further understand the function of Irr in *Brucella* iron metabolism.

## Material and methods

### Bacterial strains culture condition and Growth Kinetics Assay

*B*. *melitensis* M5-90 was donated by the Center of Chinese Disease Prevention and Control (Beijing, China). The *B*. *melitensis irr* mutant and *B*. *melitensis rirA* mutant have been constructed in a previous study [37]. *B*. *melitensis* M5-90 (M5-90), *B*. *melitensis* M5-90 *irr* mutant (*irr* mutant) and *B*. *melitensis rirA* (*rirA* mutant) were respectively cultured in normal, iron-sufficient or iron-deficient tryptic soy broth (TSB) (Difco, BD, USA) at 37°C and 150 rpm. TSB with 0.45 mM of the iron chelator 2,2′-dipyridyl (DIP, Sigma-Aldrich) or 50 μM FeCl_3_ (MACKLIN, Shanghai, China) was used as an iron-deficient or iron-sufficient medium, respectively. These two strains were monitored over a 44 h period at 37°C and 150 rpm. The absorbance value of the cell suspension was adjusted to 0.001 in a 20 μL volume and inoculated into 20 mL of TSB. The absorbance value was measured at 600 nm wavelength every 4 h for 44 h with a Nanodrop 2000 spectrophotometer (Thermo, USA). Additionally, the quantification of colony-forming units/mL (CFU/mL) for these three strains was performed using McFarland turbidimeter (BEIJING HELI KECHUANG TECHNOLOGY DEVELOPMENT CO.LTD, Beijing, China), and bacterial CFU were monitored in triplicate at 4 h intervals throughout the incubation period. All experiments with *Brucella* strain were performed in a Biosafety level 3 facility according to the regulations of Center for Disease Prevention and Control (CDC) of China.

### Iron utilization assay

The iron utilization rates of M5-90, the *irr* mutant and the *rirA* mutant in normal TSB, iron-deficient TSB, or iron-sufficient TSB were measured via 1, 10-phenanthroline chelation with ferrous iron, yielding an orange-red color complex with maximum absorption at 512 nm [38]. Briefly, during the growth kinetics assay of these three strains, 2 mL cell suspension of M5-90, *irr* mutant and *rirA* mutant from the different iron concentrations of TSB was collected at each time point and centrifuged at 12,000 × *g* for 5 min. The supernatant was transferred into a new tube, and 600 μL of 1, 10-phenanthroline (5 nM, Sigma-Aldrich) and 400 μL of L-ascorbate (0.5 mM, Sigma-Aldrich) were added and incubated for 2 h at 25°C. Finally, the absorbance value was measured at 512 nm wavelength with a Nanodrop 2000 spectrophotometer (Thermo, USA).

### Wheat cell-free protein expression

Details of wheat cell-free reaction have been described previously [39]. Cell-free protein expression was performed using the TNT SP6 Wheat Germ Master Mix Kit (Promega, Madison, WI). The primers for the irr gene are as follows: *irr* forward, 5΄-CCATATGATGCATTCTTCACATACCCA-3΄; *irr* reverse, 5΄-CTCTAGATCAGCGGGCCTGACGGCG-3΄. The polymerase chain reactions (PCRs) were incubated at 95°C for 5 min. Then 35 cycles were performed as follows: 30 s at 95°C, 40 s at 57°C, and 7 min at 72°C. The amplified PCR product was subcloned into a pDAP-Halo-Kan vector (Zoobio, Nanjing, China) using the Seamless cloning kit (Beyotime, Shanghai, China), and the recombinant plasmid was used as a transcription template. The protein expression volume was 50 μL, comprising 30 μL Wheat Germ Master Mix, 1 μg pDAP-Halo-Kan-irr plasmid, and nuclease-free water to make 50 μL; this was incubated at 25°C for 2 h. The expressed protein was confirmed using western blot analysis and an anti-Halo tag antibody (Promega, Madison, WI).

### DNA Affinity Purification Sequencing (DAP-Seq)

The genomic DNA (gDNA) of M5-90 was extracted using a Genomic DNA Extraction Kit (Zoobio, Nanjing, China). Purified gDNA was fragmented to 100–500 bp via sonication for 17 min at 20% amplitude and keeping it for 30 s on and 30 s off on ice. The size of the fragmented gDNA was checked using 1.5% agarose gel. DNA end-repair and dA-tailing were performed on 1 μg of fragmented gDNA via the NEXTflex Rapid DNA-Seq Kit (Zoobio, Nanjing, China). Ligation of end-repaired and adenylated DNA to DAP-adaptor (Mich Scientific, Beijing, China) was performed using the NEXTflex TM Enzyme Mix (Zoobio, Nanjing, China) according to the manufacturer´s instructions.

Dap-seq experiments were performed in duplicate, and beads in negative control group were incubated without protein. Ten microliters of Halo-tag magnetic beads (Yeasen, Shanghai, China) were washed three times in a 1.5 mL tube with 600 μL of sterilized PBS containing 0.01% Tween 20 (binding buffer). Twenty-five microliters of wheat cell-free expressed protein was added to 25 μL of binding buffer (containing washed beads) and incubated for 1 h on a rotating wheel at 25°C. Next, 50 μL of binding buffer was added and used to wash the bead-protein complexes five times. The supernatant was discarded, 25 μL of binding buffer (containing 100 μM MnCl_2_) and an equal volume of the gDNA library were added, and then the mixture was placed on a rotating wheel for further incubation at 25°C for 1 h. A volume of 50 μL of binding buffer was added and used to wash the formed bead-protein-DNA complexes five times to remove unbound DNA. Finally, 30 μL of 50 mM Tris-HCl, with pH 8.5 was added to suspend the complexes, and incubated for 10 min at 98°C. After incubation, the samples were cooled at 4°C for 5 min, removing the beads, and the released DNA was collected and stored at -20°C for PCR amplification as described previously [40]. The sample was sequenced using a different indexed pair of primers.

### DAP-seq data analysis

The DNA sequencing was performed using the Illumina Hiseq 2500 sequencer, and 150-bp pair-end reads were generated; all the reads were aligned to the genome of M5-90 using Bowtie2 (version 2.3.4.2), and then peak calling was conducted using MACS2 (version 2.1.1.20160309). Peaks of two duplicate samples were merged using Irreproducible Discovery Rata (IDR, v2.0.2), and the reliability of the repeated peaks was scored. The conserved motif of the region of the peaks was analyzed using MEME (v5.3.0) and the annotation of the peaks was completed using HOMER (v4.11). The distribution frequency of the reads near the transcriptional start site was analyzed using deepTools (v3.3.1).

### Quantitative real-time PCR

The expression levels of the genes identified in the Dap-seq experiment were further analyzed using quantitative real-time PCR (qRT-PCR). Total RNA was extracted from M5-90 or M5-90 *irr* mutant cells with TRIzol (CWBIO, Beijing, China). Each group has three replicates. Briefly, the RNA concentration and quality were evaluated using a Nanodrop 2000 spectrophotometer (Thermo Fisher, USA). The extracted RNA was reverse-transcribed to cDNA using a First Strand cDNA Synthesis Kit (Beyotime, Shanghai, China) according to the manufacturer’s protocol. qRT-PCR was performed using SYBR (CWBIO, Beijing, China) and a ThermoFisher QuantStudio 3 RT PCR-Well Q3 (Thermo Fisher, USA). The primers for the target genes are listed in S1 Table. The reaction conditions used are as follows: initial denaturation at 95°C for 5 min followed by 46 cycles of 95°C for 30 s and annealing at 56°C or 60°C for 30 s. The data were normalized according to the expression level of the 16S ribosomal RNA and the expression level of each gene was calculated using the 2^-ΔΔCT^ method.

### Electrophoretic Mobility Shift Assay (EMSA)

The DNA probes containing either wild type or mutated promoters of the candidate target genes were labeled using EMSA probe biotin labeling kit (Beyotime, Shanghai, China); in addition, unlabeled DNA probes were used for competition assays. Specific primers of the promoters of the target genes were listed in S2 Table. EMSAs were performed according to the manufacture´s instruction (Beyotime, Shanghai, China). Briefly, biotin-labeled DNA probes were incubated at 0.5 nM for 5 min at 37°C in an EMSA buffer (10 mM Tris-HCl, 50 mM KCl, 10 mM MgCl_2_, 10% glycerol, 0.1 mg/mL BSA, and pH 8) containing 25 ng/μL poly(dI-dC). In addition, 100 nM unlabeled DNA probes (200-fold excess and containing 100 μM MnCl_2_) were incubated with the labeled probes for competition assays. A total of 0.5 μg (100 nmol/L) Irr protein was added and incubated for an additional 15 min at 37°C. The samples were separated on a 6% non-denaturing SDS-PAGE gel and run at 100 V and 4°C in cold 0.5 × Tris-borate buffer. The DNA bands were detected using BeyoECL Plus.

### Statistical analysis

Graph plotting and all the data analyses were performed using R (v4.0.5) with the “ggplot2” package and the unpaired Student’s *t* test. All performed experiments were were repeated at least three times and the results are presented as the mean ± standard deviation. Asterisks in the figures indicate significance (* *P* < 0.05, ** *P* < 0.01, *** *P* < 0.005, and ns = not significant).

## Results

### Growth Kinetics Assays of M5-90 and the *irr* mutant in different iron concentration media

**Fig 1:**
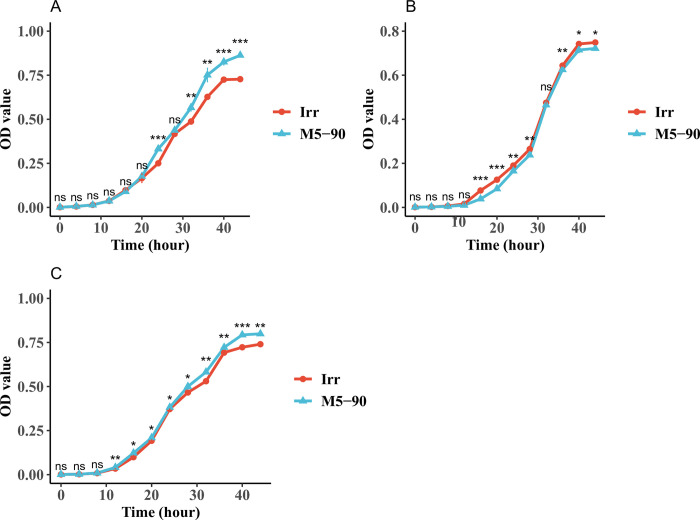
Growth curves of M5-90 and M5-90 *irr* mutant grown in (A) normal TSB, (B) iron-limited TSB, and (C) iron-sufficient TSB. The asterisk positioned atop each time point denotes the statistically significant contrast in growth between M5-90 and M5-90 *irr* mutant across various temporal intervals. The iron concentration in normal, iron-limited or sufficient TSB were 3.42 μg/mL, 3.07 μg/mL, 27.68 μg/mL. The standard deviation value in many time points cannot be presented, because the value is less than 1%.

Many studies have reported that Irr is pivotal for iron metabolism and is involved in heme biosynthesis under iron-limited conditions [41–44]. Hence, to investigate the effect of *irr* on *Brucella* growth *in vitro*, M5-90 and the *irr* mutant were grown in normal, iron-limited, and iron-sufficient TSB, and the absorbance values were measured at each time point. For the normal TSB, the growth rate of the *irr* mutant reduced significantly from 32–44 h, compared with that of the M5-90 strain (Fig 1A). However, the growth rate of the *irr* mutant was higher than that of the M5-90 strain from 16–28 h and 36–44 h under iron-limited conditions (Fig 1B). Under iron-normal or sufficient conditions, both strains exhibited a comparable growth pattern. However, it is noteworthy that the M5-90 strain demonstrated a higher growth rate in comparison to the *irr* mutant strain (Fig 1A and 1C). Additionally, the quantification of CFU/mL or CFUs for these two strains was performed under varying iron conditions at multiple time points. The results were obtained regarding CFU/mL or CFUs were found to be similar with those obtained from absorbance measurements (S1 and S2 Figs). These results suggest that *irr* can significantly affect *Brucella* growth, especially under iron-limited concentrations.

### Iron utilization assays of M5-90 and the *irr* mutant in different iron concentration media

**Fig 2:**
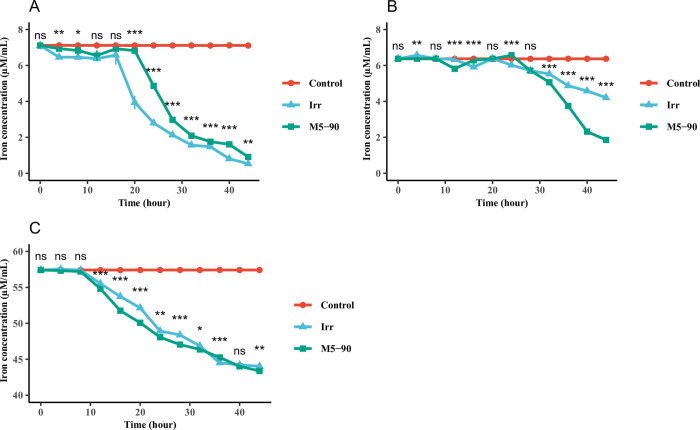
Iron utilization assays of M5-90 and M5-90 *irr* mutant grown in (A) normal TSB, (B) iron-limited TSB, and (C) iron-sufficient TSB. The iron concentration in normal, iron-limited or sufficient TSB were 7.11 μM/mL (3.42 μg/mL), 6.37 μM/mL (3.07 μg/mL), 57.4 μM/mL (27.68 μg/mL). The standard deviation value in many time points cannot be presented, because the value is less than 1%.

To further understand whether the influence of *irr* on *Brucella* growth was completed via iron utilization, M5-90 and the *irr* mutant were grown in normal TSB, iron-limited, and iron-sufficient TSB. The residual iron in the medium was determined at each time point. For the normal TSB, the iron utilization of the M5-90 was lower than that of *irr* mutant from 20–44 h (Fig 2A), whereas the iron utilization of the M5-90 was significantly higher than that of *irr* mutant from 32–44 h under iron-limited conditions (Fig 2B). Under iron-sufficient conditions, the iron utilization of the M5-90 was higher than that of *irr* mutant from 12–44 h (Fig 2C). These results indicate that *irr* can affect *Brucella* growth via iron utilization, especially under iron-limited condition.

### Identification of Irr-bound DNA Sequences from the *Brucella* genome

**Fig 3:**
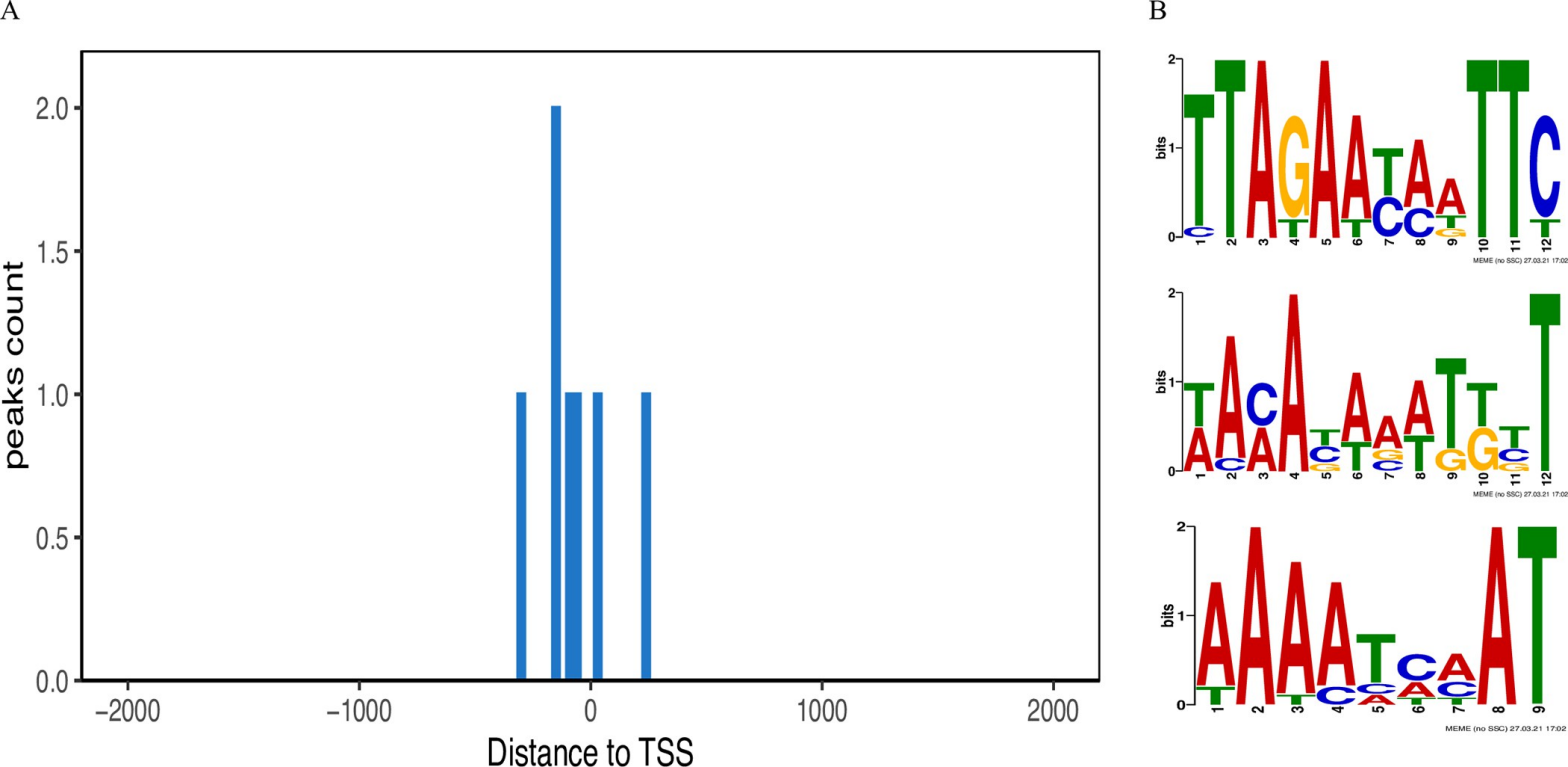
Data generated by Dap-seq. (A) Peaks generated by Dap-seq. The regions of Irr-bound DNA produced seven peaks when mapped to the *B*. *melitensis* M5-90 genome. (B) Enriched DNA motifs obtained with MEME-Dap-seq on relevant Dap-seq peaks corresponding to near summit regions.

Given that Irr protein can affect growth and iron utilization of *Brucella* especially under iron-limited conditions, it is postulated that the numerous genes related to iron metabolism could be modulated by the Irr protein. Thus, to investigate the underlying mechanisms of Irr involvement in iron metabolism. The Daq-seq was used to identify the Irr-bound DNA sequences from the *Brucella* genome. The Irr protein was purified and confirmed using Western blotting (S3A Fig), and the fragmented *Brucella* genome was checked using agarose gel (S3B Fig). After DNA sequencing, aligning, and peak calling, seven peaks were generated on the Irr-bound region, and seven genes were identified in the *Brucella* genome (Fig 3A and S3 Table); of these, three genes were located in chromosome I (NC_003317.1) and four genes were located in chromosome II (NC_003318.1) (S4 Fig). In addition, three motifs of the Irr protein were identified in the *Brucella* genome (Fig 3B), which is consistent with the results obtained in previous studies [33, 45], and all of these sequences are A/T-rich. Overall, these results indicate that seven genes were identified as target genes of the Irr protein for regulating iron metabolism in *Brucella* genome.

### Identification of the expression levels of the Irr-bound genes in M5-90 and the *irr* mutant

**Fig 4:**
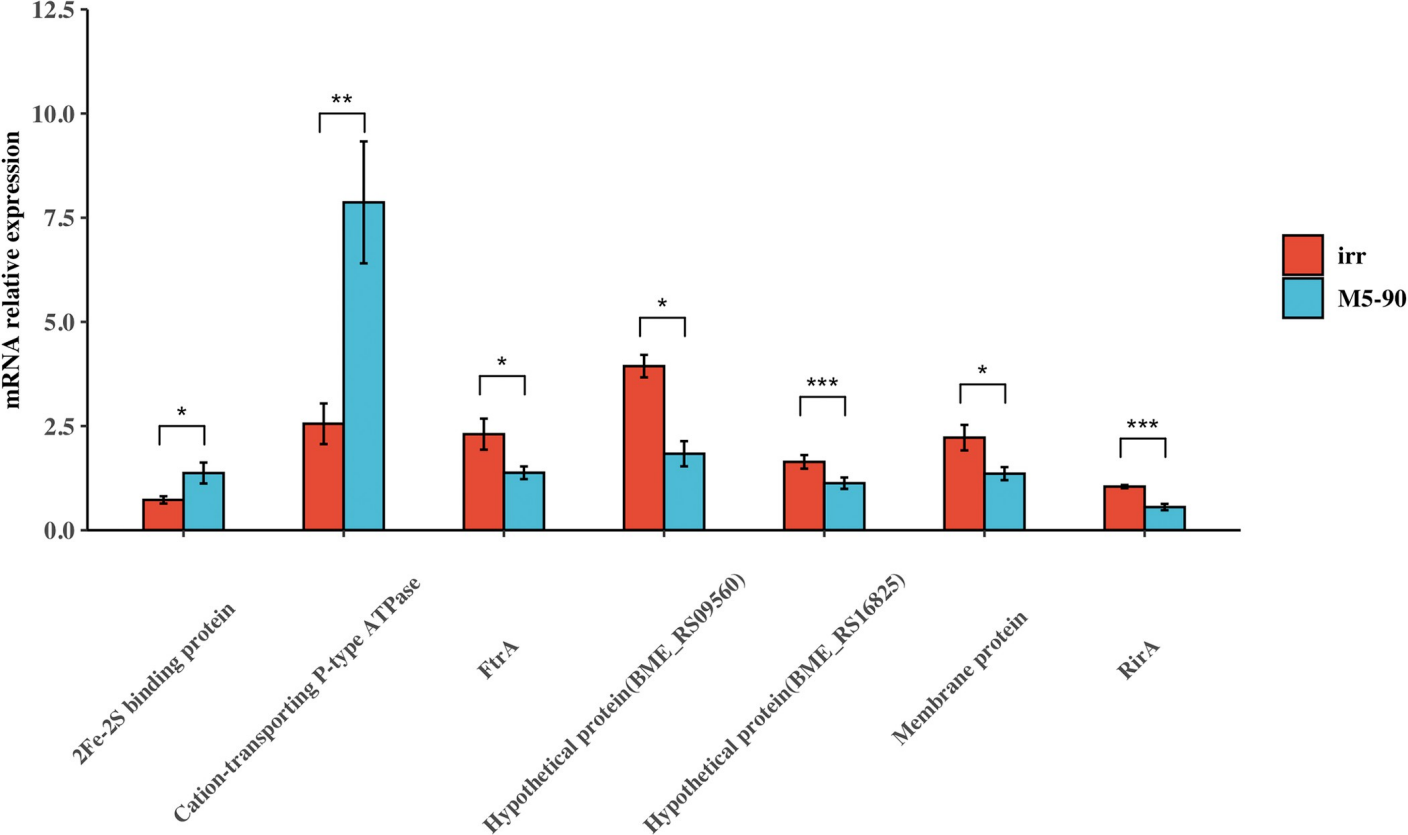
Identification of the expression levels of the seven genes using qRT-PCR. Total RNA was isolated from M5-90 or the M5-90 *irr* mutant and cDNA was synthesized. *2Fe-2S binding protein*, *Cation-transporting P-type ATPase (zntA)*, *FtrA*, *Hypothetical protein (BME_RS09560)*, *Hypothetical protein (BME_RS16825)*, *Membrane protein*, and *RirA* genes were analyzed using qRT-PCR. The expression levels of the targets gene were normalized by the expression of *16 S*. The Y-axis represents the gene expression in the M5-90 *irr* mutant or M5-90.

A total of seven Irr-bound genes have been identified previously. To further confirm the expression levels of these genes in M5-90 and the *irr* mutant, RNA was isolated from these two strains grown to log phase (32 h) in iron-limited TSB, and cDNA was synthesized. Of these seven genes, the expression levels of five genes (*BME_RS09560*, *membrane protein*, *RirA*, *Iron transporter* (*ftrA)*, and *BME_RS16825*) were higher in the *irr* mutant than in the M5-90 strain (Fig 4). The expression levels of two genes (*2Fe-2S binding protein* and *cation-transporting P-type ATPase (zntA)*) were lower in the *irr* mutant than in the M5-90 strain (Fig 4). Therefore, these seven genes were regulated by *irr* and might interact directly with the Irr protein to regulate iron metabolism.

### Confirmation of the interactions of the Irr protein with the seven genes using EMSA

**Fig 5:**
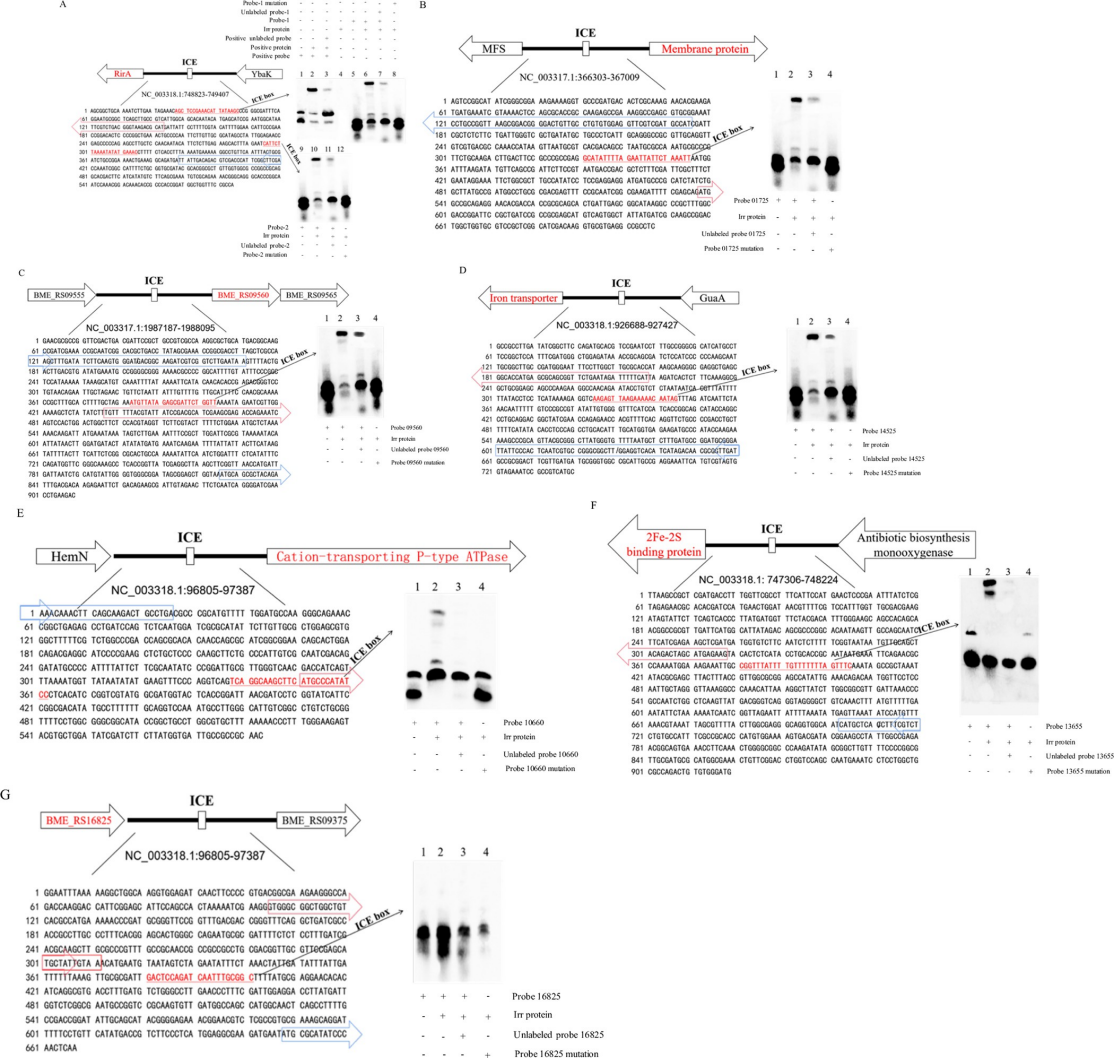
Genetic organization of the seven genes in M5-90 and the Irr protein interacts directly with the promoters of these genes in an EMSA. (A) Irr protein interacts with *BME_RS13665* (rirA). The red and blue arrows indicate *rirA* and *YbaK* gene sequences. The red and underlined sequences represent the putative Irr binding sites (ICE-box); lane 1: positive probe, lane 2: positive probe + positive protein, lane 3: positive probe + positive protein + positive unlabeled probe, lane 4: Irr protein, lane 5: probe *BME_RS13665*-1, lane 6: probe *BME_RS13665*-1 + Irr protein, lane 7: probe *BME_RS13665*-1 + unlabeled probe *BME_RS13665*-1 + Irr protein, lane 8: probe *BME_RS13665*-1 mutation + Irr protein, lane 9: probe *BME_RS13665*-2, lane 10: probe *BME_RS13665*-2 + Irr protein, lane 11: probe *BME_RS13665*-2 + unlabeled probe *BME_RS13665*-2 + Irr protein, and lane 12: probe *BME_RS13665*-2 mutation + Irr protein. (B) Irr protein interacts with *BME_RS01725* (membrane protein). The red and blue arrows indicate *BME_RS01725* and *MFS* gene sequences. The red and underlined sequences represent the putative Irr binding site (ICE-box); lane 1: probe *BME_RS01725*, lane 2: probe *BME_RS01725* + Irr protein, lane 3: probe *BME_RS01725* + unlabeled *BME_RS01725* + Irr protein, and lane 4: probe *BME_RS01725* mutation + Irr protein; (C) Irr protein interacts with *BME_RS09560* (hypothetical protein). The red and blue arrows indicate *BME_RS09560*, *BME_RS09555*, and *BME_RS09565* gene sequences. The red and underlined sequences represent the putative Irr binding site (ICE-box); lane 1: probe *BME_RS09560*, lane 2: probe *BME_RS09560* + Irr protein, lane 3: probe *BME_RS09560* + unlabeled *BME_RS09560* + Irr protein, and lane 4: probe *BME_RS09560* mutation + Irr protein. (D) Irr protein interacts with *BME_RS14525* (Iron transporter). The red and blue arrows indicate *BME_RS14525* and *GuaA* gene sequences. The red and underlined sequences represent the putative Irr binding site (ICE-box); lane 1: probe *BME_RS14525*, lane 2: probe *BME_RS14525* + Irr protein, lane 3: probe *BME_RS14525* + unlabeled *BME_RS14525* + Irr protein, and lane 4: probe *BME_RS14525* mutation + Irr protein; (E) Irr protein interacts with *BME_RS10660* (cation-transporting P-type ATPase). The red and blue arrows indicate *BME_RS10660* and *HemN* gene sequences. The red and underlined sequences represent the putative Irr binding site (ICE-box); lane 1: probe *BME_RS10660*, lane 2: probe *BME_RS10660* + Irr, lane 3: probe *BME_RS10660* + unlabeled probe *BME_RS10660* + Irr protein, and lane 4: probe *BME_RS10660* mutation + Irr protein. (F) Irr protein interacts with *BME_RS13655* (2Fe-2S binding protein). The red and blue arrows indicate *BME_RS13655* and antibiotic biosynthesis monooxygenase gene sequences. The red and underlined sequences represent the putative Irr binding site (ICE-box); lane 1: probe *BME_RS13655*, lane 2: probe *BME_RS13655* + Irr protein, lane 3: probe *BME_RS13655* + unlabeled probe *BME_RS13655* + Irr protein, and lane 4: probe *BME_RS13655* mutation + Irr protein. (G) Irr protein interacts with *BME_RS16825* (hypothetical protein). The red and blue arrows indicate *BME_RS16825* and *BME_RS09375* gene sequences. The red and underlined sequences represent the putative Irr binding site (ICE-box); lane 1: probe *BME_RS16825*, lane 2: probe *BME_RS16825* + Irr protein, lane 3: probe *BME_RS16825* + unlabeled probe *BME_RS16825* + Irr protein, and lane 4: probe *BME_RS16825* mutation + Irr protein.

To further verify the interactions of the Irr protein and the seven genes, EMSA was performed to determine whether the Irr protein can directly bind to these seven genes. As shown in Fig 5, the promoter regions of six out of the seven genes were able to form protein-DNA complexes with the Irr protein, and these genes are *RirA* (BME_RS13665, gene ID: 29595065), *membrane protein* (BME_RS01725, gene ID: 29593120), *hypothetical protein* (BME_RS09560, gene ID: 29594811), *FtrA* (BME_RS14525, gene ID: 29595411), *cation-transporting P-type ATPase* (*zntA*) (BME_RS10660, gene ID: 29595149), and *2Fe-2S binding protein* (BME_RS13655, gene ID: 29595949). Collectively, these results indicate that the Irr protein can directly bind to *rirA*, *membrane protein*, *hypothetical protein*, *ftrA*, *zntA*, and *2Fe-2S binding protein* to participate in iron metabolism in *Brucella*.

### Iron utilization assays of M5-90 and the *rirA* mutant in different iron concentration media

**Fig 6:**
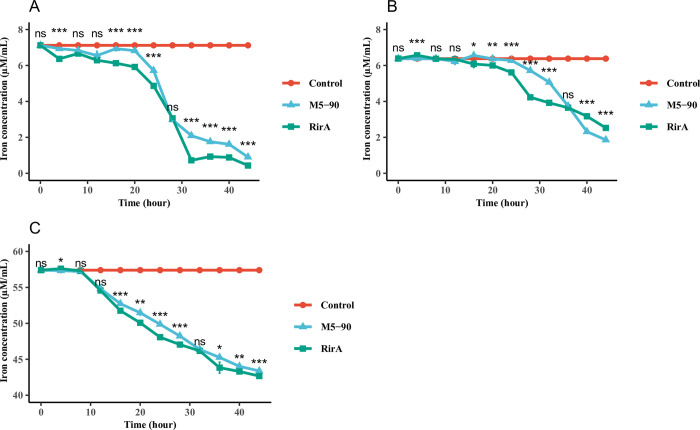
Iron utilization assays of M5-90 and M5-90 *rirA* mutant grown in (A) normal TSB, (B) iron-limited TSB, and (C) iron-sufficient TSB. The iron concentrations in normal, iron-limited or sufficient TSB were 7.12 μM/mL (3.43 μg/mL), 6.38 μM/mL (3.08 μg/mL), 57.4 μM/mL (27.68 μg/mL). The standard deviation value in many time points cannot be presented, because the value is less than 1%.

The RirA is another iron response regulator and it also involved in iron uptake and energy metabolism [35,43]. Hence, to further investigate the role of *rirA* in iron metabolism of *Brucella*, M5-90 and the *rirA* mutant were cultured in normal TSB, iron-limited, and iron-sufficient TSB. The residue iron in the medium was determined at each time point. For the normal TSB, the iron utilization of the M5-90 was lower than that of *rirA* from 16–24 h and 32–44 h (Fig 6A); under iron-limited conditions, the iron utilization of the M5-90 was lower than that of *rirA* from 16–32 h, whereas, the iron utilization of the M5-90 was higher than that of *rirA* from 40–44 h (Fig 6B). In order to elucidate the dissimilar phenotype observed in M5-90 under iron-depleted conditions, as compared to the *rirA* mutant strain, an investigation was conducted into the expression of *irr* and *rirA* genes during mid-exponential, late-exponential and stationary phases of growth. Results showed a progressive enhancement in gene expression of both *irr* and *rirA* from 16 h to 40 h. While the expression of *rirA* started to decline after 40 h and till 45 h, there was a rapid increase in the expression of *irr* during the same time frame. (S5 Fig). In addition, the iron utilization of the *rirA* was higher than that of M5-90 from 16–28 h and 36–44 h (Fig 6C). Collectively, these results suggested that *rirA* can affect iron metabolism of *Brucella* at different iron concentration conditions, especially under iron-sufficient and normal conditions, these two strains have similar iron metabolism pattern.

### Growth kinetics assays of M5-90 and the *rirA* mutant in different iron concentration media

**Fig 7:**
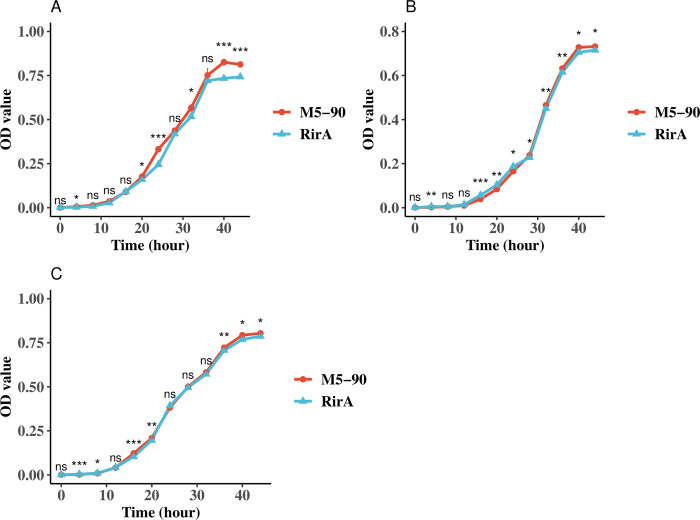
Growth curves of M5-90 and M5-90 *rirA* mutant grown in (A) normal TSB, (B) iron-limited TSB, and (C) iron-sufficient TSB. The asterisk positioned atop each time point denotes the statistically significant contrast in growth between M5-90 and M5-90 *irr* mutant across various temporal intervals. The iron concentrations in normal, iron-limited or sufficient TSB were 3.43 μg/mL, 3.08 μg/mL, 27.68 μg/mL. The standard deviation value in many time points cannot be presented, because the value is less than 1%.

Previous study reported that iron is a fundamental micronutrient for *Brucella*, and plays a role in its growth [46]. Therefore, to investigate whether the *rirA* can affect *Brucella* growth via iron utilization, M5-90 and the *rirA* mutant were cultured in normal TSB, iron-limited, and iron-sufficient TSB. The absorbance values were measured at each time point. For the normal TSB, the growth rate of the *rirA* was lower than that of the M5-90 from 20–24 h and 40–44 h (Fig 7A). However, the growth rate of the *rirA* mutant was higher than that of the M5-90 from 16–24 h, and the growth rate of the *rirA* mutant was lower than that of the M5-90 from 28–44 h under iron-limited conditions (Fig 7B). For the iron-sufficient conditions, the growth rate of the *rirA* was lower than that of the M5-90 from 16–20 h and 36–44 h (Fig 7C). In addition, the quantification of CFU/mL or CFUs for these two strains was performed under varying iron conditions at multiple time points. The results were obtained regarding CFU/mL or CFUs were found to be similar with those obtained from absorbance measurements (S6 and S7 Figs). Overall, these results demonstrated that the *rirA* can affect *Brucella* growth at different iron concentration conditions via iron utilization.

## Discussion

Iron is required by all life forms owing to its involvement in many fundamental biological processes, including photosynthesis, N_2_ fixation, methanogenesis, H_2_ production and consumption, respiration, the trichloroacetic acid (TCA) cycle, oxygen transport, gene regulation, and DNA biosynthesis [47]. *B*. *melitensis* is a Gram-negative intracellular pathogen that causes abortion and infertility in livestock. As with other bacteria, iron is an essential micronutrient for *Brucella*, and plays a role in its growth and virulence [46,48]. However, maintaining iron homeostasis is also crucial for *Brucella*, because reactive oxygen can be produced in an iron-replete environment, which is not in favor of the survival of *Brucella*. Several studies have suggested that Irr is a major iron response regulator in many species and participates in heme biosynthesis [28,30,49]. The expression of the Irr protein is controlled by cellular iron levels, and is degraded under iron-sufficient conditions when heme is biosynthesized [30,32,42]. However, the Irr protein is activated under iron-limited conditions and represses heme biosynthesis [30]. Heme is a key iron source for *Brucella* growth, therefore, it might be the reason for the higher growth rate of the *irr* mutant than that of M5-90 under iron-limited conditions (Fig 1B). Under normal or iron-sufficient conditions, the Irr protein is less expressed or degraded. Remarkably, the growth rate of M5-90 surpasses that of the *irr* mutant under both normal and iron-sufficient circumstances (Fig 1A and 1C). This leads to the inference that the *irr* may exert an impact on the growth of *Brucella* in normal or iron-sufficient conditions through elusive mechanisms, despite being in iron-sufficient conditions, the mutation of *irr* was found to alter the expression of various genes associated with iron metabolism. Nonetheless, the underlying mechanisms responsible for this phenomenon warrant further investigation. In addition, the mutation of *irr* reduced the iron utilization of *B*. *melitensis* under iron-limited conditions (Fig 2B), and that phenotype may be produced by the Irr protein interacting with *zntA* or *2Fe-2S binding protein* identified in this study (Fig 5E and 5F). The expression levels of these two genes were significantly reduced when *irr* was mutated (Fig 4). However, the M5-90 strain and *irr* mutant have different patterns of iron utilization under normal or iron-sufficient conditions (Fig 2A and 2C). The reason for that phenomenon may be attributed to the characteristics of the Irr protein, and it can serve as either a repressor or an activator of many genes based on the cellular iron level [43,44]. Despite the degradation of Irr protein under iron-sufficient conditions, the expression of other genes involved in iron metabolism can still be affected by *irr*, potentially influencing the growth rate and iron utilization of *Brucella*.

**Fig 8:**
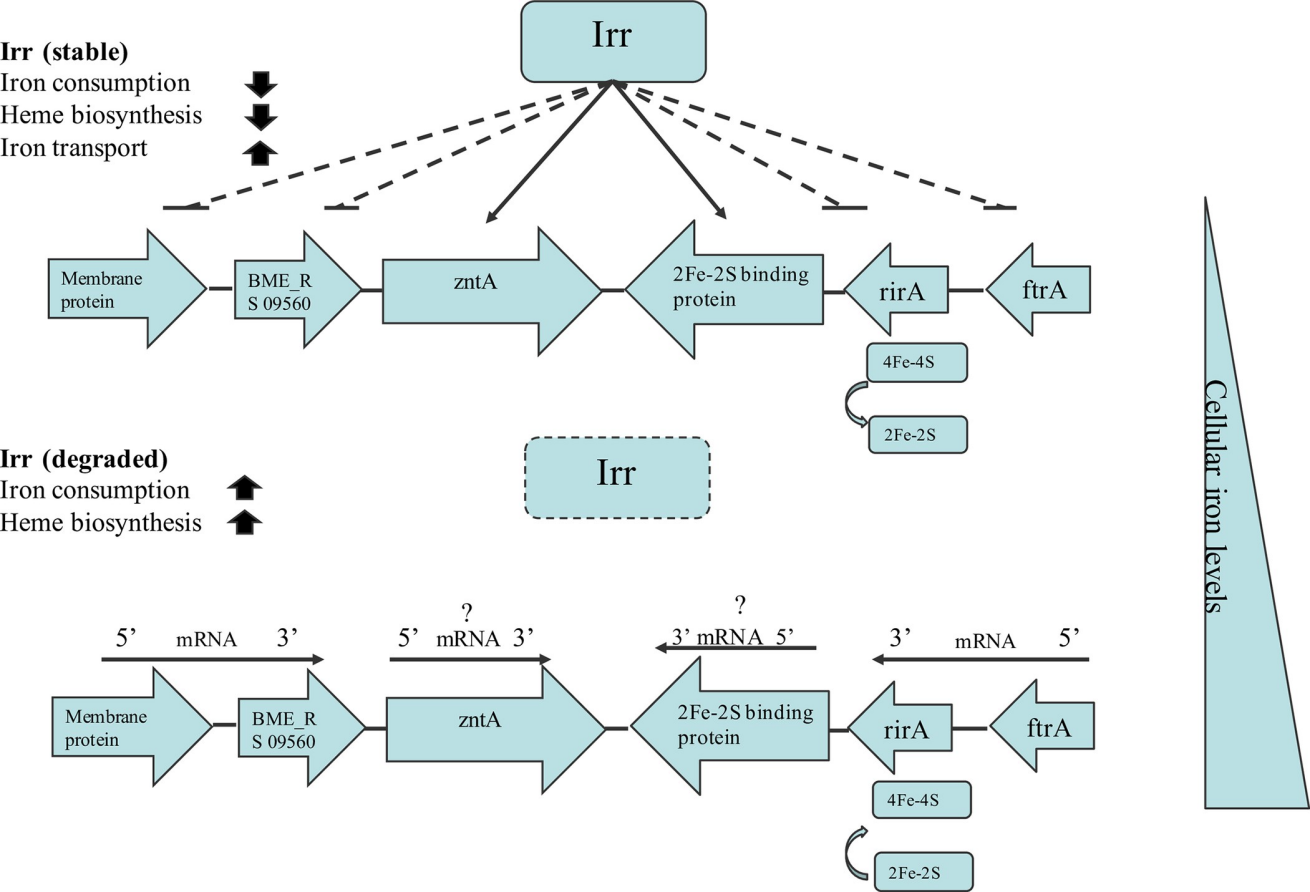
Model showing the proposed role of Irr and RirA in *Brucella* iron metabolism. Under iron-limited conditions, Irr remains stable and exerts a repressive effect on the expression of genes responsible for iron consumption or heme biosynthesis to ensure the maintenance of essential processes. On the other hand, Irr promotes the expression of genes attributed to iron transport. In iron-sufficient conditions, the degradation of Irr allows the expression of multiple genes associated with iron consumption and heme biosynthesis. When intracellular iron levels exceed a certain threshold, the 2Fe-2S clusters within RirA undergo conversion into 4Fe-4S clusters, resulting in the suppression of genes involved in iron uptake.

The RirA protein is another iron response regulator and was initially discovered in *Rhizobium leguminosarum* [35]. RirA participates in many physiological processes, such as iron uptake, energy metabolism, and heme biosynthesis [43]. RirA can be activated under iron-sufficient conditions to inhibit the iron uptake system. However, under iron-limited conditions, the expression of RirA was repressed by Irr to reduce iron consumption for maintaining essential processes in *Agrobacterium tumefaciens* [36]. In this study, the mutation of the *rirA* resulted in a significant enhancement of iron uptake of *Brucella*, especially under iron normal or -sufficient conditions, which further corroborated that the RirA can be activated under iron-sufficient conditions to inhibit iron uptake for maintaining iron homeostasis. In addition, the higher iron utilization of *rirA* mutant might be the reason for its lower growth rate under iron normal or -sufficient conditions, because the excessive iron uptake was not benefit for the growth of *Brucella* [46]. Whereas, the iron utilization of *rirA* mutant was not consistent under iron-limited conditions, the iron utilization of the *rirA* mutant was higher than that of the M5-90 from 16–32 h, but was lower than that of the M5-90 from 36–44 h. Additionally, the growth rate of the *rirA* mutant was also inconsistent under iron-limited conditions, whereas, the underlying mechanisms responsible for the observed phenotype may potentially be linked to the fluctuating expression patterns of *irr* or *rirA*, which in turn be influenced by the iron concentration within the medium. In this work, the expression level of *rirA* (BME_RS13665) was higher in the *irr* mutant than that in the M5-90 strain (Fig 4), and the promoter of the *rirA* can form a complex with the Irr protein (Fig 5A). Hence, Irr can directly inhibit *rirA* expression under iron-limited conditions, which is consistent with the results obtained from *Agrobacterium tumefaciens* [36]. Furthermore, the RirA contains the mixture of both [2Fe–2S] and [4Fe–4S] forms, and the [4Fe–4S] can inhibit the expression of many genes involved in iron uptake; however, under low iron conditions, the [4Fe–4S] was converted to the [2Fe–2S] to alleviate the repression effect [50]. Therefore, the Irr might regulate the functions of RirA via the conversion reaction from [4Fe–4S] to [2Fe–2S] for maintaining iron homeostasis under iron limited conditions (Fig 8). The FtrA have been identified a periplasmic iron-binding protein and act as a ferrous iron transporter in *Brucella* [51]. The non-haem iron utilization of *Brucella* was reduced when the *ftrA* gene was mutated and the iron-responsive expression of *ftrA* was dependent on the *irr* under iron-limited conditions. Interestingly, the *ftrA* (BME_RS14525) was also identified in our results, however, the expression of the *ftrA* in this work was contrary to the results obtained from a previous study [51]. The expression of *ftrA* was enhanced when *irr* was mutated in this work. This difference may be attributed to the iron concentration in TSB, and the iron concentration in the iron-deficient medium may not reach a threshold to trigger the *irr* to promote the expression of *ftrA* for transporting ferrous iron. Whereas, further studies are required to validate this hypothesis.

The MbfA is an inner membrane protein identified as a key iron exporter in *B*. *japonicum* [52]. The *mbfA* mutant displayed severely defective in iron export activity and contains more than two folds of intracellular iron level than the parent strain. Additionally, it has been described that the *mbfA* can be repressed by Irr under iron-limited conditions for iron homeostasis [53,54]. In the current study, we discovered that the sequence identity of the *mbfA* and the *membrane protein* (BME_RS01725) is 65%, hence, it can be inferred that these two genes may have some similar functions. Additionally, the expression of *membrane protein* was also inhibited by *irr* under iron-limited conditions and the Irr protein can directly bind to the promoter of *membrane protein* for iron regulation.

P-type ATPases make up a superfamily of transport proteins that are mediated by ATP hydrolysis [55]. Inorganic cations are mainly substrates for these proteins, including K^+^, Na^+^, Mg^2+^, Ca^2+^ and Zn^2+^[56]. Each P-type ATPase can import its substrates from the periplasm to the cytoplasm or export it from cytoplasm to the periplasm. Experimental evidence suggested that the *zntA* is a Zn-specific exporter that can export Zn^2+^ out of the cell, which prevents the toxicity of Zn^2+^ in *Brucella* [18]. In our results, the *zntA* was also identified as a Irr regulon in DAP-seq. Hence, the Irr protein might also involve in Zn^2+^ transport for maintaining zinc level in *Brucella*. In addition to *rirA* and *ftrA*(*iron transporter*), three other genes, namely, *membrane protein*, *hypothetical protein and 2Fe-2S binding protein*, could also bind to the Irr protein. However, the specific functions relate to these genes remain unknown in *Brucella* and it is also the limitation of this study. Additionally, the Irr protein can also bind to the promoter regions of *dhbCEBA*, *bhuA*, and *bhuQ* to regulate iron metabolism in *Brucella* [31–33]. However, these genes were not identified in our study, this discrepancy may be related to the different iron concentrations under the experimental conditions, because the expression of *bhuA* and *bhuQ* was determined by iron concentration or *irr* per se, specifically, the expression of *Brucella abortus* 2308 *bhuA* was enhanced under iron-limited conditions, but was reduced under iron-sufficient conditions or when *irr* was mutated [32].

In summary, seven genes related to iron metabolism were identified in this study, and six out of the seven genes can interact with the Irr protein and may participate in iron metabolism. It is also important to determine the role of *membrane protein* (BME_RS01725), *hypothetical protein* (BME_RS09560) and *2Fe-2S binding protein* (BME_RS13655) in *Brucella* iron metabolism. In addition, the *rirA* was identified as a target gene for Irr and also participated in iron metabolism and *Brucella* growth. Overall, the results obtained in this study provide valuable information for the exploration of *Brucella* iron metabolism.

## Supporting information

S1 Fig Growth curves of M5-90 and M5-90 *irr* mutant grown in (A) normal TSB, (B) iron-limited TSB, and (C) iron-sufficient TSB. The asterisk positioned atop each time point denotes the statistically significant contrast in growth between M5-90 and M5-90 *irr* mutant across various temporal intervals.
(TIF)

S2 Fig The number of CFUs of M5-90 and M5-90 *irr* mutant grown in (A) normal TSB, (B) iron-limited TSB, and (C) iron-sufficient TSB. The asterisk positioned atop each time point denotes the statistically significant contrast in growth between M5-90 and M5-90 *irr* mutant across various temporal intervals.
(TIF)

S3 Fig Confirmation of cell-free expressed Irr protein using western blot analysis and identification of fragmented genome of *B*. *melitensis* M5-90. (A) Western blot analysis of Irr protein; 20 μg of the Irr protein was separated using 4–12% SDS-page and immunoblotted with anti-Halo tag antibody. The anticipated molecule mass (51 kDa) of Irr is shown; lane M: Protein Marker (Sangon Biotech), lane 1: Irr protein; (B) Agarose gel analysis of fragmented *Brucella* genome; lane M: Marker B (Sangon Biotech), lane 1: fragmented *Brucella* genome.
(TIF)

S4 Fig The distribution of peaks in the chromosome. The height represents the quality of peaks.
(TIF)

S5 Fig The gene expression of *irr* and *rirA* was determined at mid- exponential, late-exponential and stationary culture phases of M5-90 in iron-limited conditions. Total RNA was isolated from M5-90 and cDNA was synthesized. The expression levels of the targets gene were normalized by the expression of *16 S*.
(TIF)

S6 Fig Growth curves of M5-90 and M5-90 *rirA* mutant grown in (A) normal TSB, (B) iron-limited TSB, and (C) iron-sufficient TSB. The asterisk positioned atop each time point denotes the statistically significant contrast in growth between M5-90 and M5-90 *irr* mutant across various temporal intervals.
(TIF)

S7 Fig The number of CFUs of M5-90 and M5-90 *rirA* mutant grown in (A) normal TSB, (B) iron-limited TSB, and (C) iron-sufficient TSB. The asterisk positioned atop each time point denotes the statistically significant contrast in growth between M5-90 and M5-90 *irr* mutant across various temporal intervals.
(TIF)

S1 Table The primers used for qRT-PCR. (DOCX)

S2 Table The primers used in EMSA experiments. (DOCX)

S3 Table The seven sequences identified in Dap-seq. (XLSX)

S4 Table The raw data of qRT-PCR for identification of the expression levels of the seven genes. (CSV)

S1 Data The raw data of the Dap-seq replica_1. (CSV)

S2 Data The raw data of the Dap-seq replica_2. (CSV)
