# Supplementary figures and images for: Scanning iron response regulator binding sites using Dap-seq in the Brucella genome
Source: PLoS Negl Trop Dis. 2023 Jul 17;17(7):e0011481. doi: 10.1371/journal.pntd.0011481 (PMC10374146; doi:10.1371/journal.pntd.0011481)

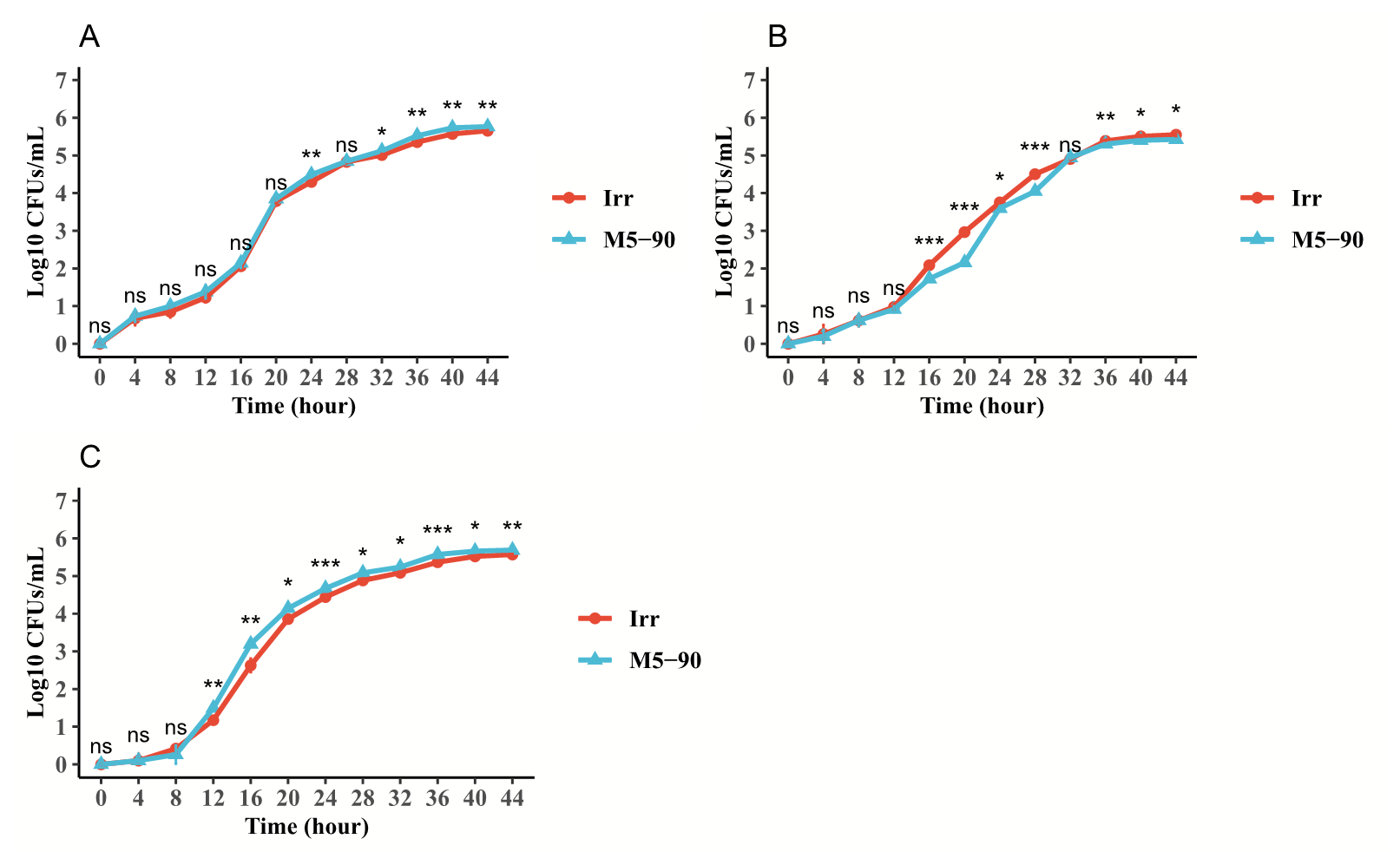

Supplement: S1 Fig — Growth curves of M5-90 and M5-90 irr mutant grown in (A) normal TSB, (B) iron-limited TSB, and (C) iron-sufficient TSB. The asterisk positioned atop each time point denotes the statistically significant contrast in growth between M5-90 and M5-90 irr mutant across various temporal intervals. (TIF) [file pntd.0011481.s001.tif]

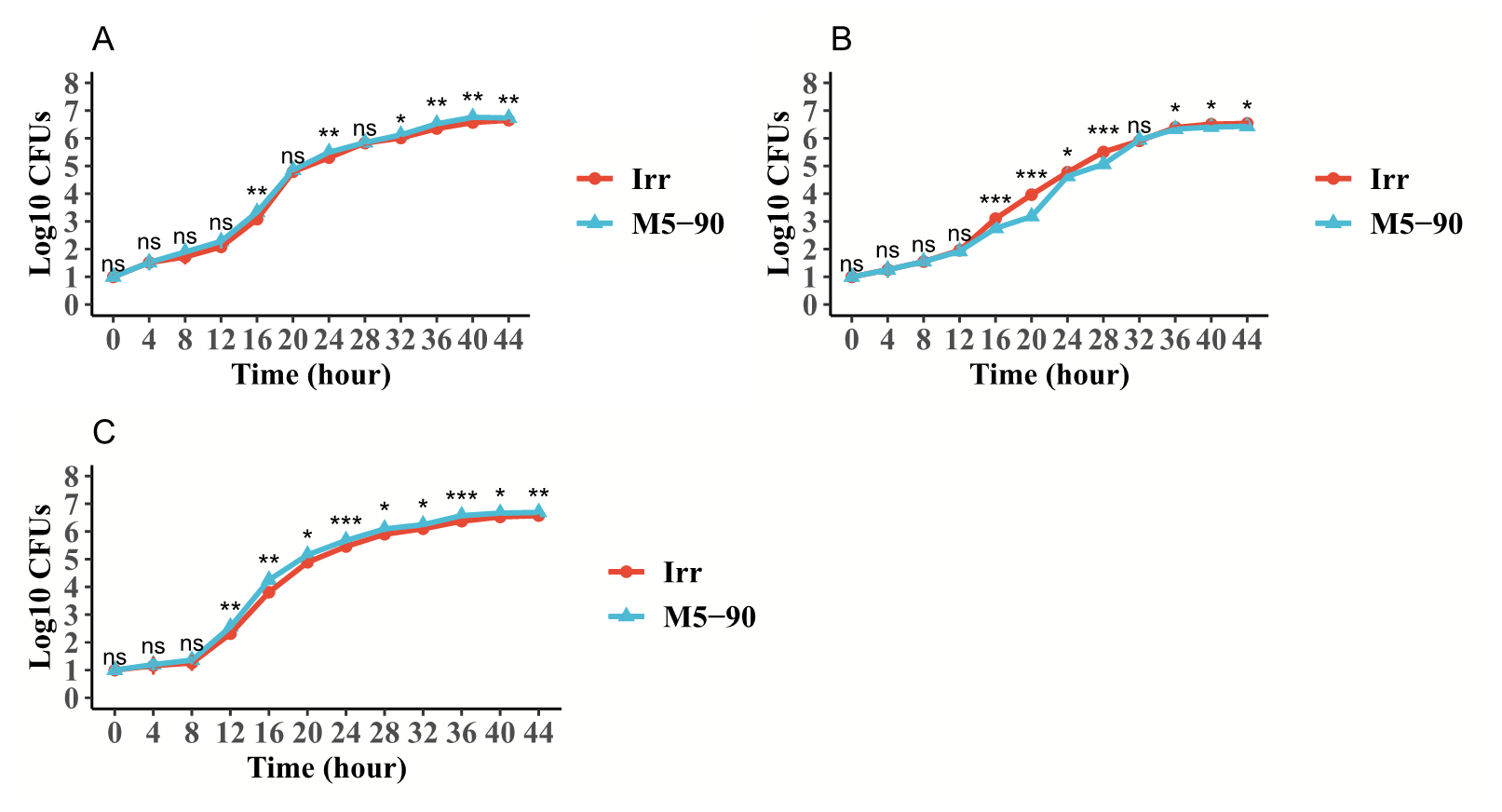

Supplement: S2 Fig — The number of CFUs of M5-90 and M5-90 irr mutant grown in (A) normal TSB, (B) iron-limited TSB, and (C) iron-sufficient TSB. The asterisk positioned atop each time point denotes the statistically significant contrast in growth between M5-90 and M5-90 irr mutant across various temporal intervals. (TIF) [file pntd.0011481.s002.tif]

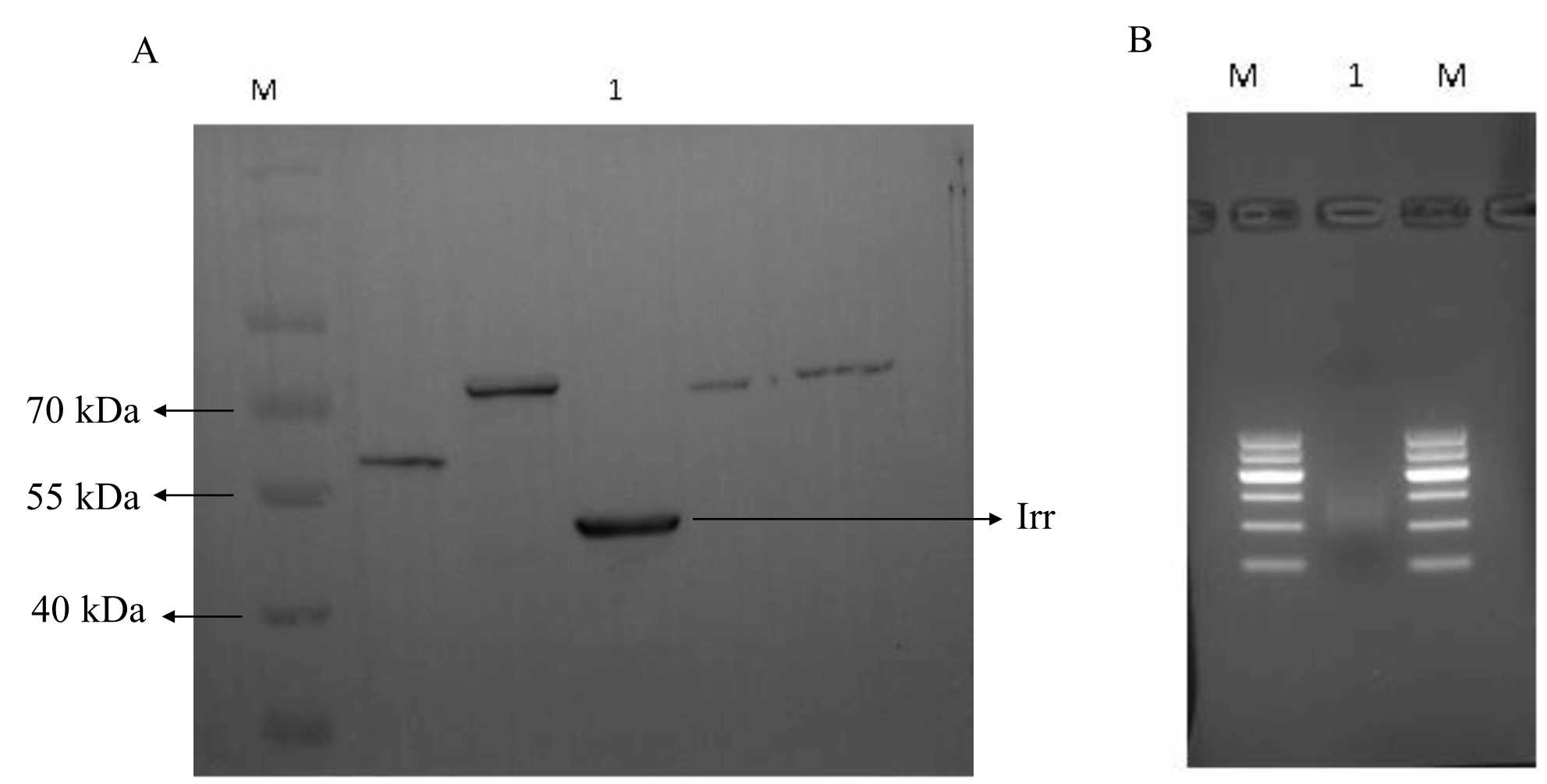

Supplement: S3 Fig — (A) Western blot analysis of Irr protein; 20 μg of the Irr protein was separated using 4–12% SDS-page and immunoblotted with anti-Halo tag antibody. The anticipated molecule mass (51 kDa) of Irr is shown; lane M: Protein Marker (Sangon Biotech), lane 1: Irr protein; (B) Agarose gel analysis of fragmented Brucella genome; lane M: Marker B (Sangon Biotech), lane 1: fragmented Brucella genome. (TIF) [file pntd.0011481.s003.tif]

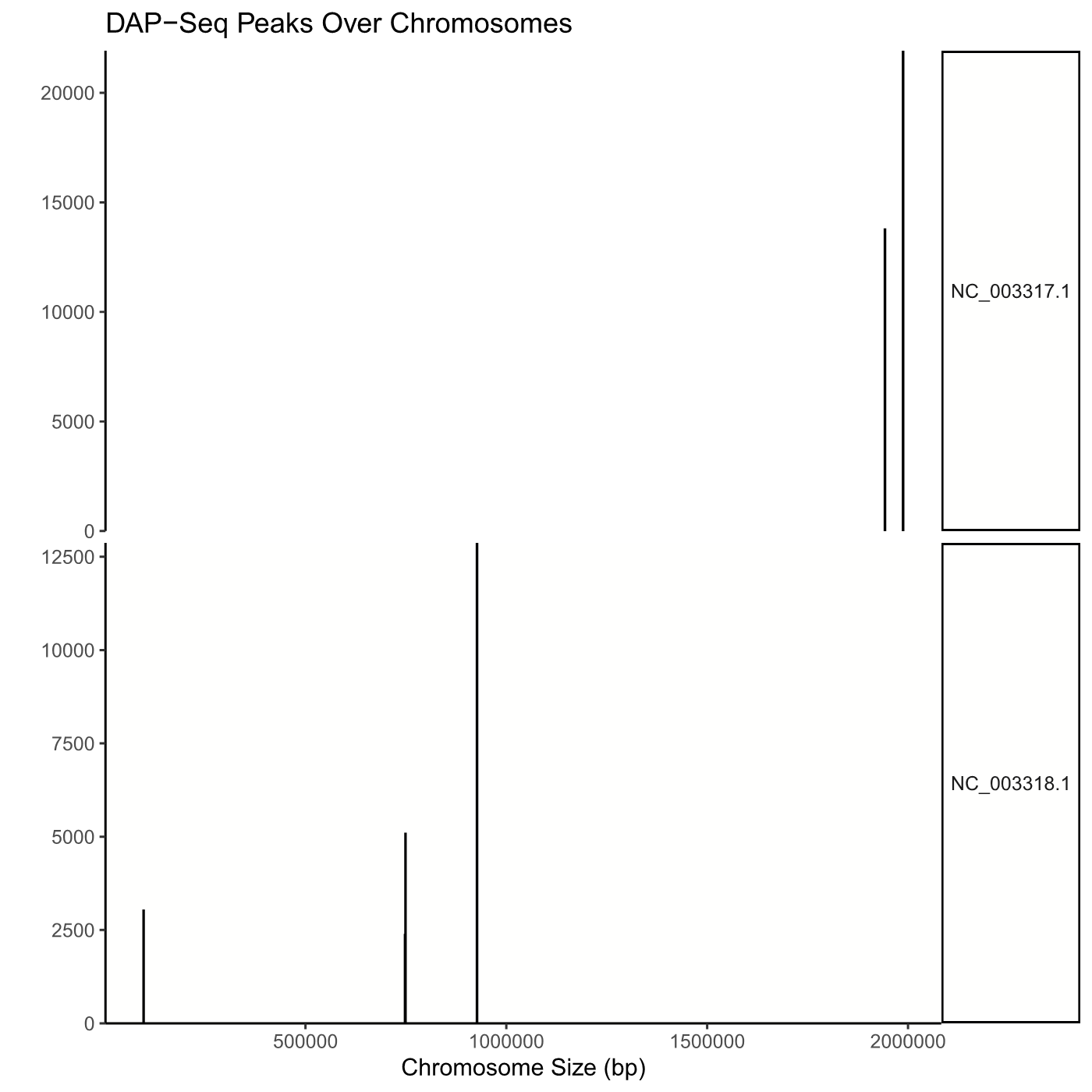

Supplement: S4 Fig — The height represents the quality of peaks. (TIF) [file pntd.0011481.s004.tif]

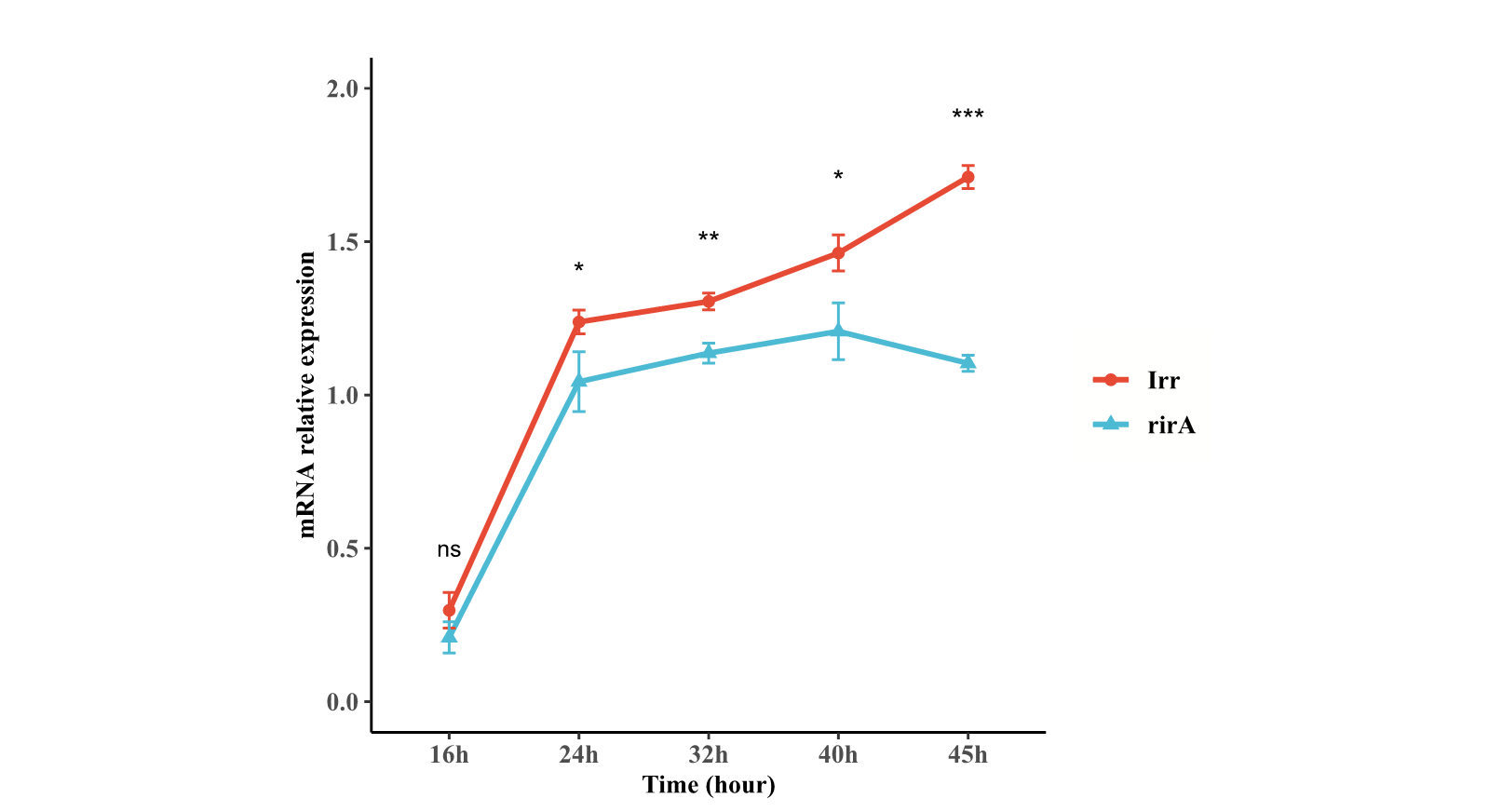

Supplement: S5 Fig — Total RNA was isolated from M5-90 and cDNA was synthesized. The expression levels of the targets gene were normalized by the expression of 16 S. (TIF) [file pntd.0011481.s005.tif]

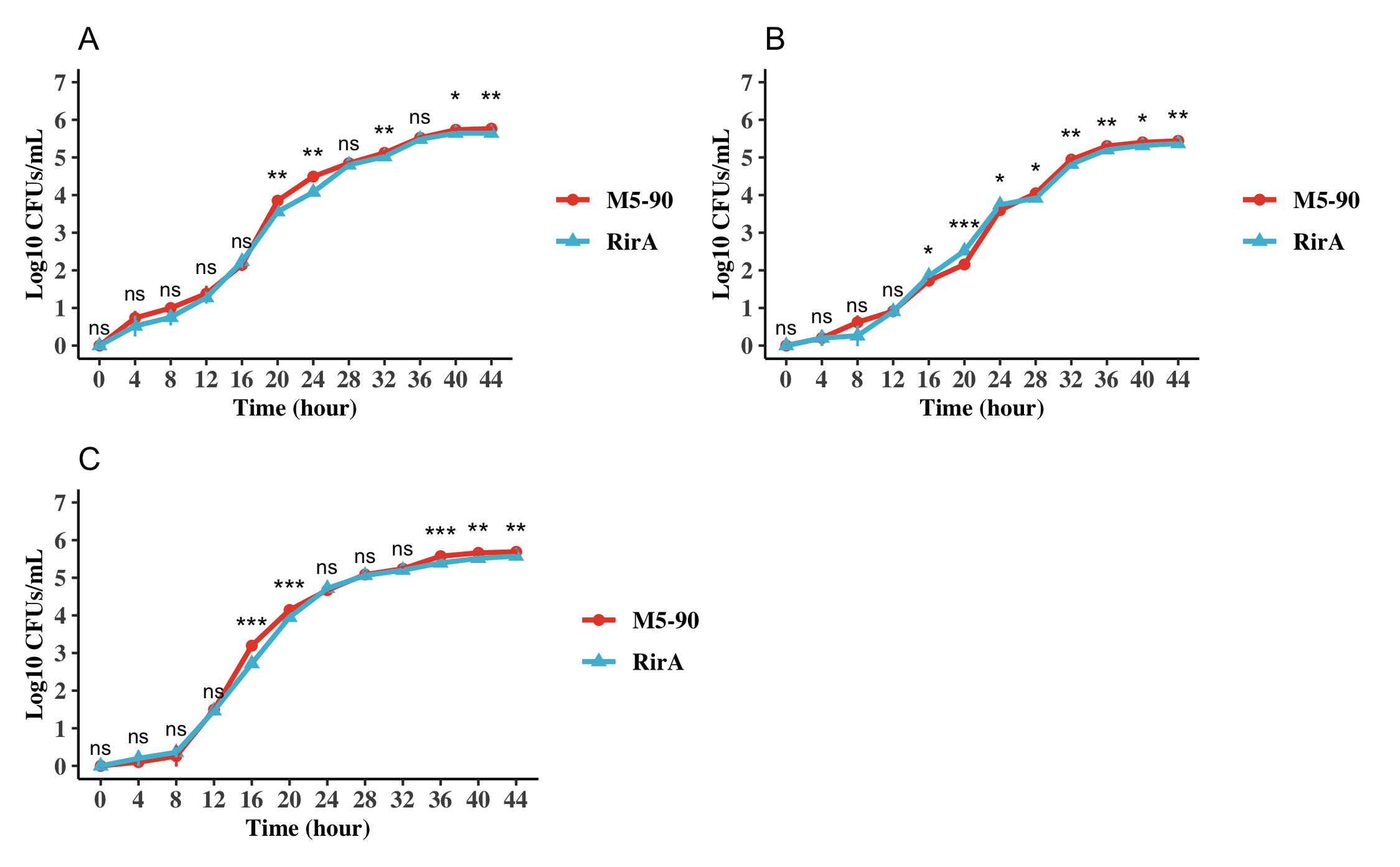

Supplement: S6 Fig — Growth curves of M5-90 and M5-90 rirA mutant grown in (A) normal TSB, (B) iron-limited TSB, and (C) iron-sufficient TSB. The asterisk positioned atop each time point denotes the statistically significant contrast in growth between M5-90 and M5-90 irr mutant across various temporal intervals. (TIF) [file pntd.0011481.s006.tif]

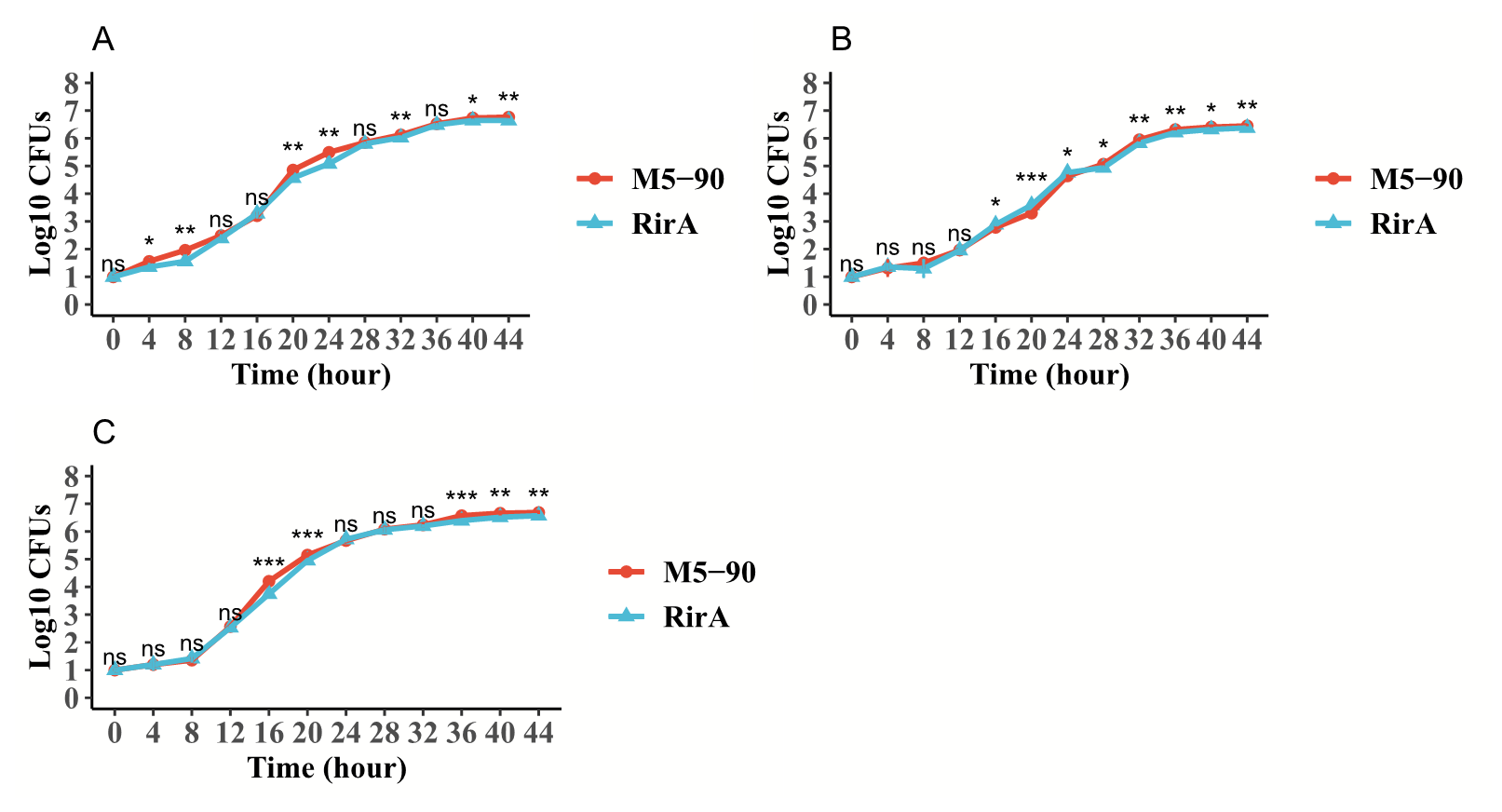

Supplement: S7 Fig — The number of CFUs of M5-90 and M5-90 rirA mutant grown in (A) normal TSB, (B) iron-limited TSB, and (C) iron-sufficient TSB. The asterisk positioned atop each time point denotes the statistically significant contrast in growth between M5-90 and M5-90 irr mutant across various temporal intervals. (TIF) [file pntd.0011481.s007.tif]
