# Supplementary material for: Scanning iron response regulator binding sites using Dap-seq in the Brucella genome
Source: PLoS Negl Trop Dis. 2023 Jul 17;17(7):e0011481. doi: 10.1371/journal.pntd.0011481 (PMC10374146; doi:10.1371/journal.pntd.0011481)
Supplement: S1 Table — (DOCX) [file pntd.0011481.s008.docx]

**Supporting Table 1.** Primers used for qRT-PCR.

| Gene names | Primer sequences (5’-3’) |
| --- | --- |
| *16 S*-F | CACTGGACCATTACTGACGC |
| *16 S*-R | ACTAAGGGCGAGGGTTGC |
| *BME_RS09560*-F | TTTTACTTTCATTCTCGGCGCACTG |
| *BME_RS09560*-R | GAGGCTTTGCCCGAACCATCTG |
| *RirA-*F | AGCTGAGCCGCATTCCTGAAATC |
| *RirA*-R | ACCGTTTCCACCAATCCGTGTTC |
| *2Fe-2S binding protein-*F | TGCTGTGGCTGCTTCCCAAATG |
| *2Fe-2S binding protein-R* | AGAACGCACACGATCCATGAACTG |
| *BME_RS16825*-F | GGTTTGACGACCGGGTTTCAGG |
| *BME_RS16825*-R | AGCTTGCGTCGATCAAAGGAGAG |
| *membrane protein-*F | AAGGATTTCTGGTCTGCGACAAGC |
| *membrane protein-*R | TAGCGGTTCTGAATGAAGGCAATGG |
| *Iron transporter-*F | GCGGTTTATCTCCAGCCCATCG |
| *Iron transporter-R* | CACCCAGTCACCTTCGGCAAAG |
| *Irr-* F | AGTTTACGGAAGCGGGCATGTTG |
| *Irr-* R | GCTGGTGATCGGAGATATTGGTGTC |
| *cation-transporting P-type ATPase-*F | CGGATCAGCCACGCATCAGTTC |
| *cation-transporting P-type ATPase-R* | GACGGTCGCCTGTAAGCATCAC |
