# Supplementary material for: Scanning iron response regulator binding sites using Dap-seq in the Brucella genome
Source: PLoS Negl Trop Dis. 2023 Jul 17;17(7):e0011481. doi: 10.1371/journal.pntd.0011481 (PMC10374146; doi:10.1371/journal.pntd.0011481)
Supplement: S2 Table — (DOCX) [file pntd.0011481.s009.docx]

**Supporting Table 2.** Primers used in EMSA

| Gene names | Primer sequences（5’-3’） |
| --- | --- |
| *BME_RS13665*-1-F | CATTCTTAAAATATATGAAAC |
| *BME_RS13665*-1-R | GTTTCATATATTTTAAGAATG |
| *BME_RS13665*-1-unlabeled-F | CATTCTTAAAATATATGAAAC |
| *BME_RS13665*-1 mutation-F | CATTCTTGGGGCCCGGGAAAC |
| *BME_RS13665*-1 mutation-R | GTTTCCCGGGCCCCAAGAATG |
| *BME_RS13665*-2-F | AGGAATAATCATGCGTCTTAC |
| *BME_RS13665*-2-R | GTAAGACGCATGATTATTCCT |
| *BME_RS13665*-2-unlabeled-F | AGGAATAATCATGCGTCTTAC |
| *BME_RS13665*-2 mutation-F | AGGGGCGGCAGCGCGTCTTAC |
| *BME_RS13665*-2 mutation-R | GTAAGACGCGCTGCCGCCCCT |
| *BME_RS01725*-F | GCATATTTTAGAATTATTCTAAATT |
| *BME_RS01725*-R | AATTTAGAATAATTCTAAAATATGC |
| *BME_RS01725*-unlabeled-F | GCATATTTTAGAATTATTCTAAATT |
| *BME_RS01725* mutation-F | GCATATTTCGTGGCCGCCACAAATT |
| *BME_RS01725* mutation-R | AATTTGTGGCGGCCACGAAATATGC |
| *BME_RS14525*-F | CTTAATGAAAAATCTATTCAG |
| *BME_RS14525*-R | CTGAATAGATTTTTCATTAAG |
| *BME_RS14525*-unlabeled-F | CTTAATGAAAAATCTATTCAG |
| *BME_RS14525* mutation-F | CTTAATGAGGGGCACGCTCAG |
| *BME_RS14525* mutation-R | CTGAGCGTGCCCCTCATTAAG |
| *BME_RS09560*-F | ATGTTATAGAGCGATTCTGGTT |
| *BME-RS09560*-R | AACCAGAATCGCTCTATAACAT |
| *BME_RS09560*-unlabeled-F | ATGTTATAGAGCGATTCTGGTT |
| *BME_RS09560* mutation-F | ATGTTATGTGTATGCCACGGTT |
| *BME_RS09560* mutation-R | AACCGTGGCATACACATAACAT |
| *BME_RS13655*-F | CGGTTTATTTTGTTTTTTTAGTTTC |
| *BME_RS13655*-R | GAAACTAAAAAAACAAAATAAACCG |
| *BME_RS13655*-unlabeled-F | CGGTTTATTTTGTTTTTTTAGTTTC |
| *BME_RS13655* mutation-F | CGGTTTATGGGGGGGGGGGGGTTTC |
| *BME_RS13655* mutation-R | GAAACCCCCCCCCCCCCATAAACCG |
| *BME_RS10660*-F | TCAGGCAAGCTTCATGCCCATATCC |
| *BME_RS10660*-R | GGATATGGGCATGAAGCTTGCCTGA |
| *BME_RS10660*-unlabeled-F | TCAGGCAAGCTTCATGCCCATATCC |
| *BME_RS10660* mutation-F | TCAGGCAAAAAAAAAAAAAATATCC |
| *BME_RS10660* mutation-R | GGATATTTTTTTTTTTTTTGCCTGA |
| *BME_RS16825*-F | GACTCCAGATCAATTTGCGGC |
| *BME_RS16825*-R | GCCGCAAATTGATCTGGAGTC |
| *BME_RS16825*-unlabeled-F | GACTCCAGATCAATTTGCGGC |
| *BME_RS16825* mutation-F | GACTGGGGGGGAAAAAGCGGC |
| *BME_RS16825* mutation-R | GCCGCTTTTTCCCCCCCAGTC |

Red represents mutated DNA bases.
